# Supplementary figures and images for: SARS-CoV-2 Nsp14 mediates the effects of viral infection on the host cell transcriptome
Source: eLife. 2022 Mar 16;11:e71945. doi: 10.7554/eLife.71945 (PMC9054133; doi:10.7554/eLife.71945)

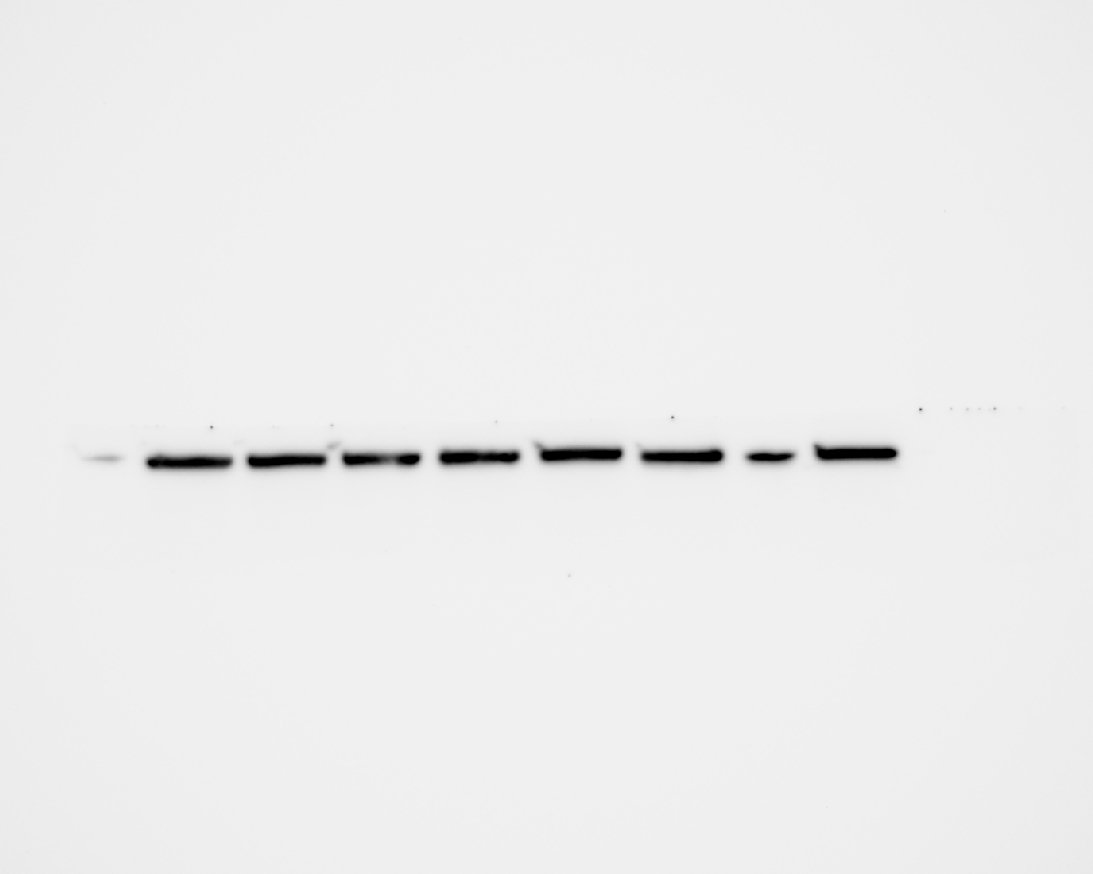

Supplement: Figure 4—source data 1. [file elife-71945-fig4-data1.zip › Figure 4 - source data 1/Figure 4 - source data 1_antiActin.jpg]

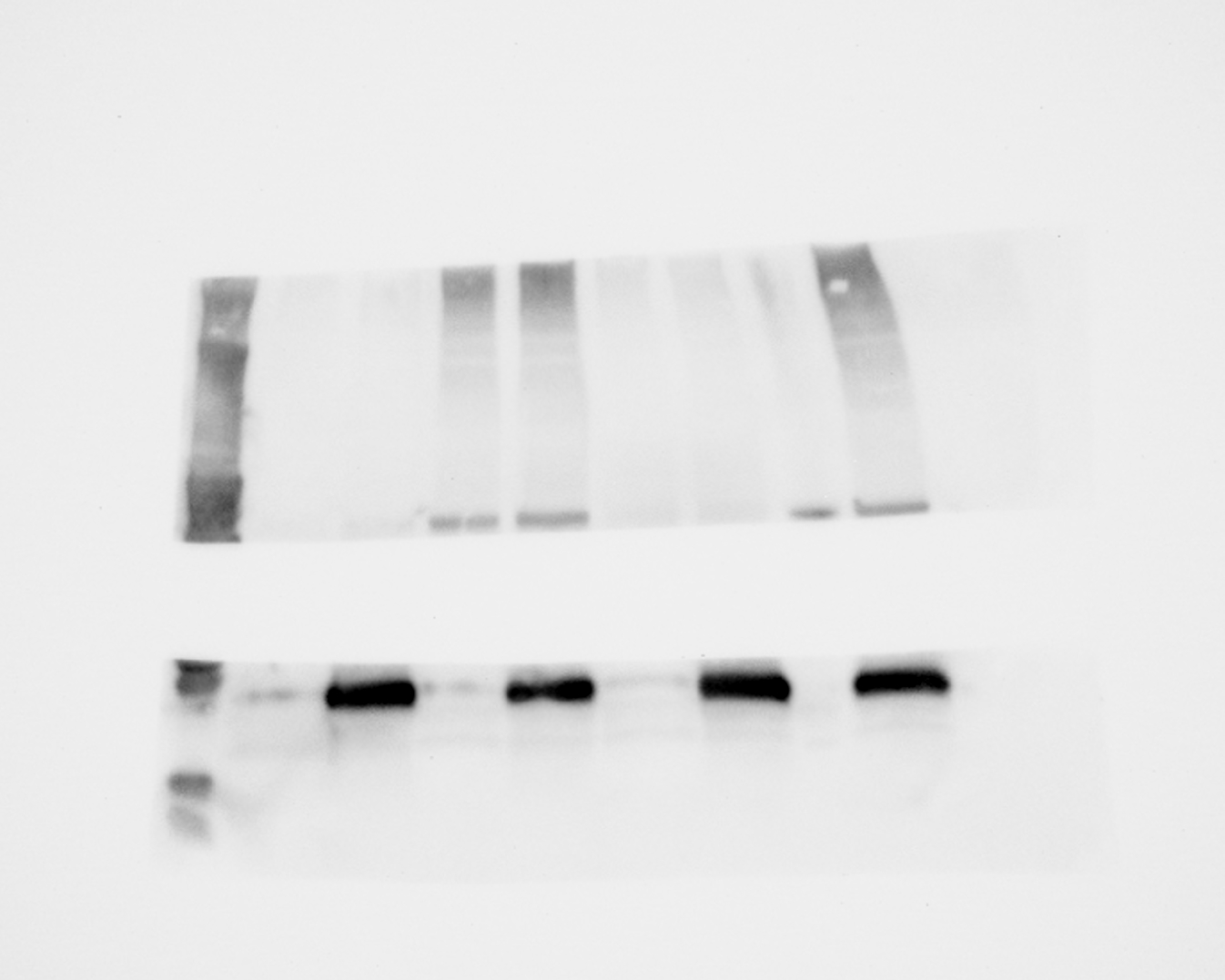

Supplement: Figure 4—source data 1. [file elife-71945-fig4-data1.zip › Figure 4 - source data 1/Figure 4 - source data1_antiStrep.jpg]

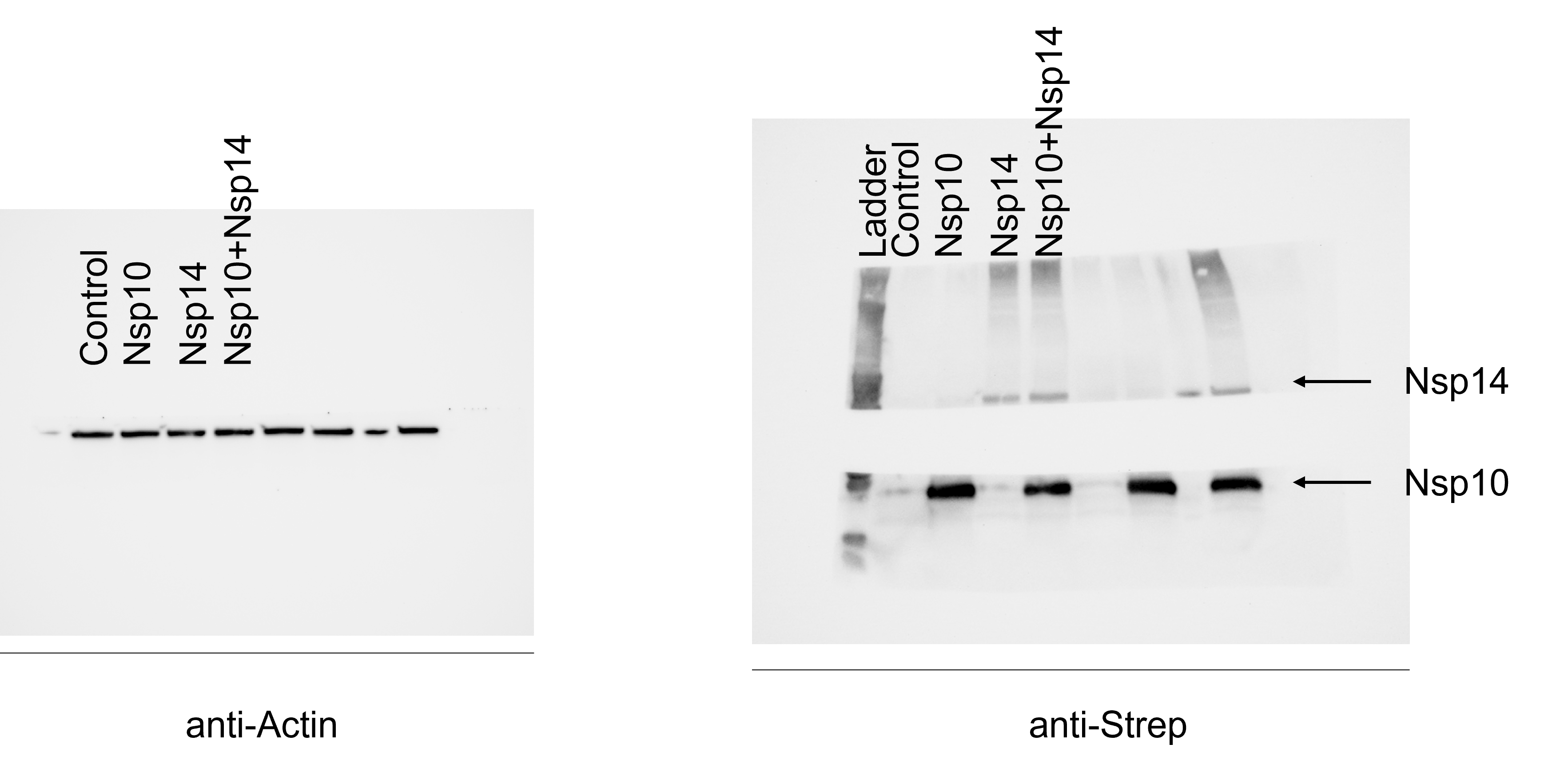

Supplement: Figure 4—source data 1. [file elife-71945-fig4-data1.zip › Figure 4 - source data 1/Figure 4 - source data1_Labelled.png]

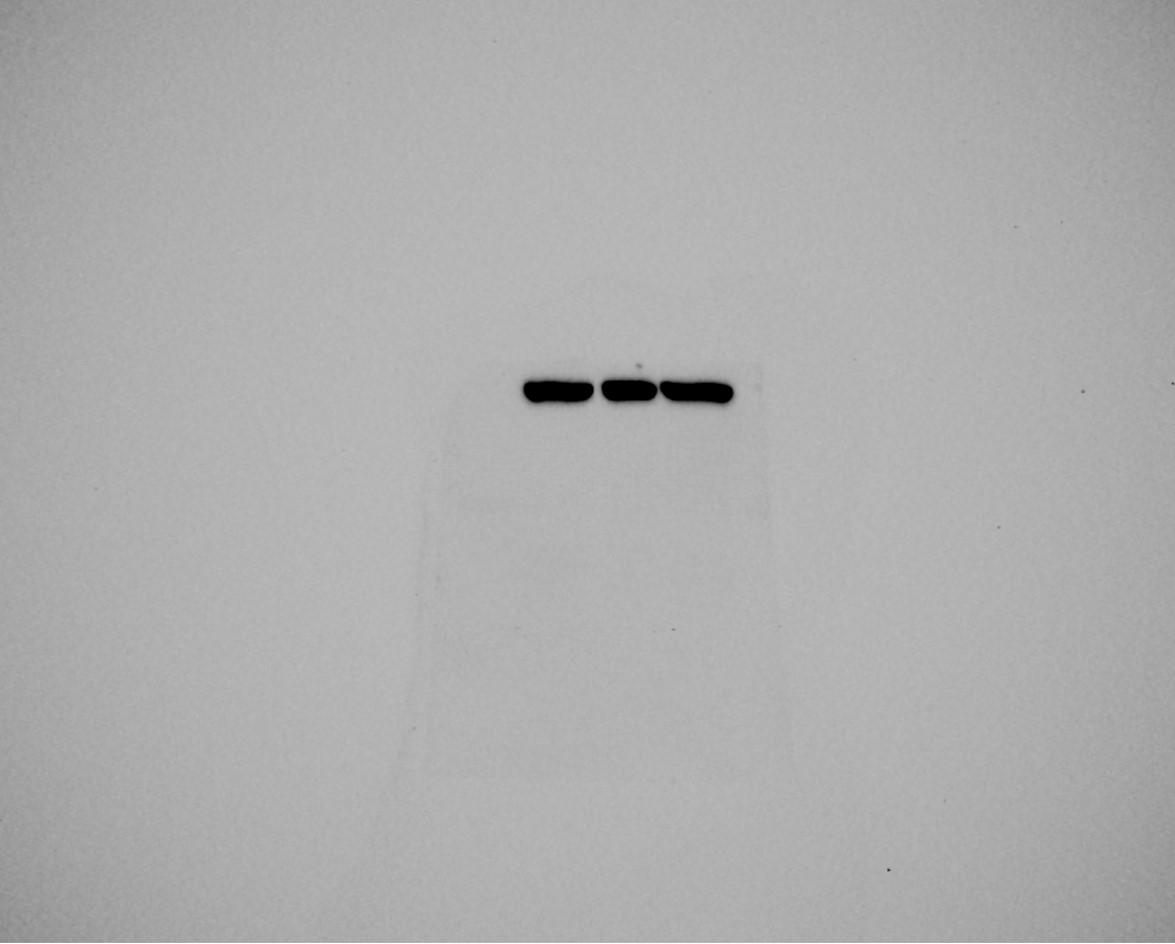

Supplement: Figure 4—figure supplement 2—source data 1. [file elife-71945-fig4-figsupp2-data1.zip › Figure 4 - figure supplement 2 - source data 1/Figure 4 -figure supplement 2 - source data 1_antiActin.jpg]

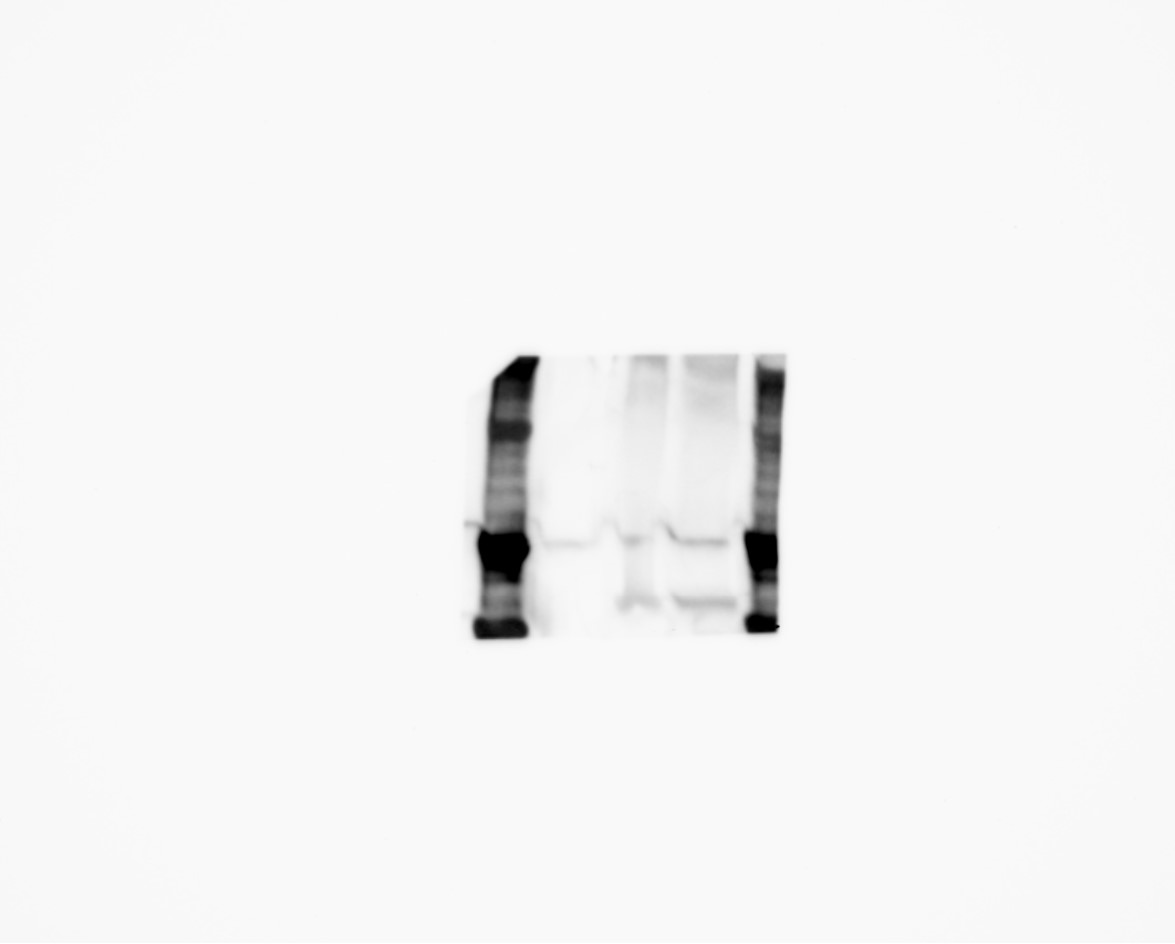

Supplement: Figure 4—figure supplement 2—source data 1. [file elife-71945-fig4-figsupp2-data1.zip › Figure 4 - figure supplement 2 - source data 1/Figure 4 -figure supplement 2 - source data 1_antiStrep.jpg]

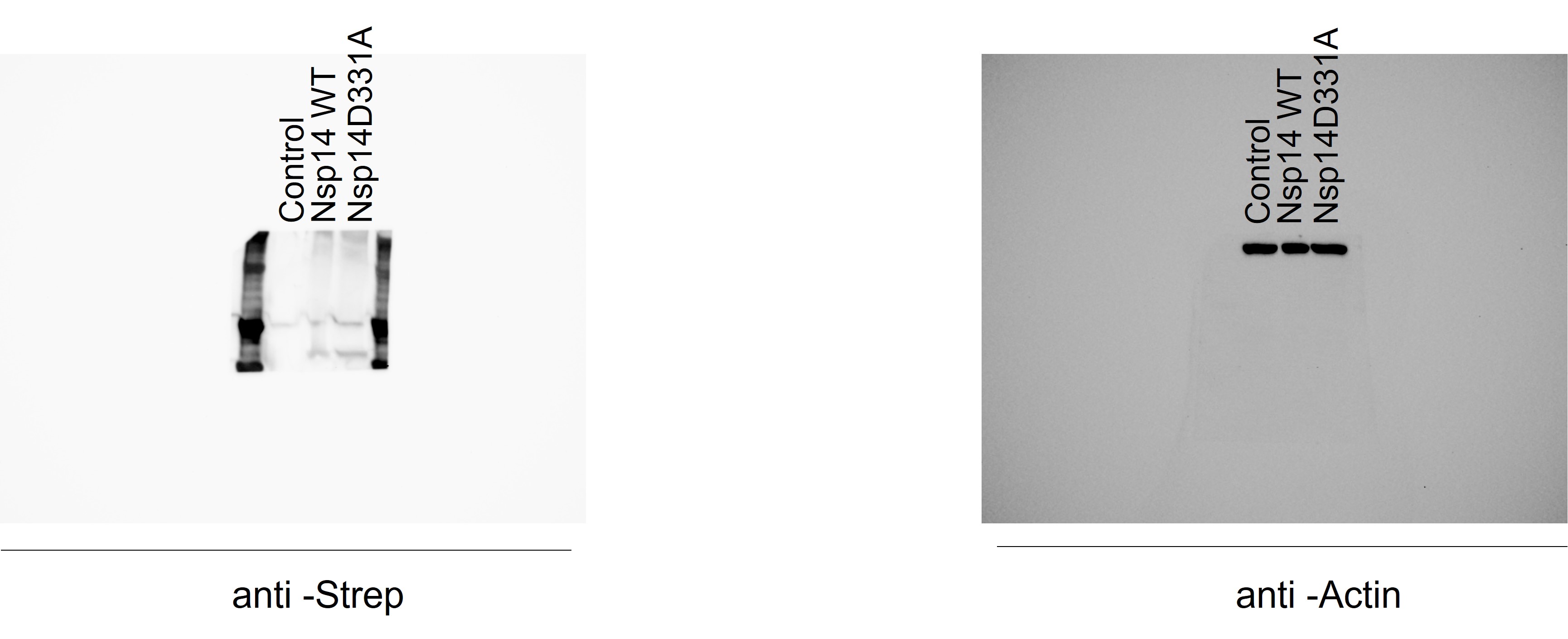

Supplement: Figure 4—figure supplement 2—source data 1. [file elife-71945-fig4-figsupp2-data1.zip › Figure 4 - figure supplement 2 - source data 1/Figure 4 -figure supplement 2 - source data 1_Labelled.jpg]

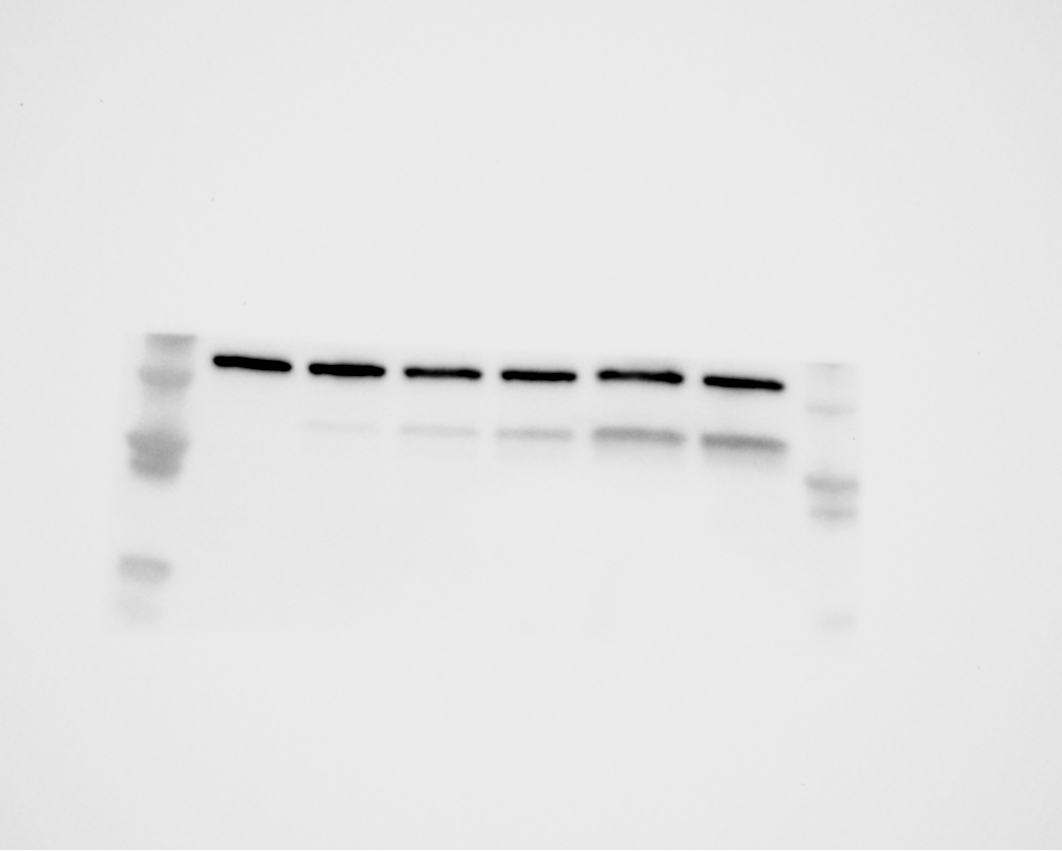

Supplement: Figure 5—figure supplement 1—source data 1. [file elife-71945-fig5-figsupp1-data1.zip › Figure 5 - figure supplement 1 - source data 1/Figure 5 - figure supplent 1 - source data 1_antiActin.jpg]

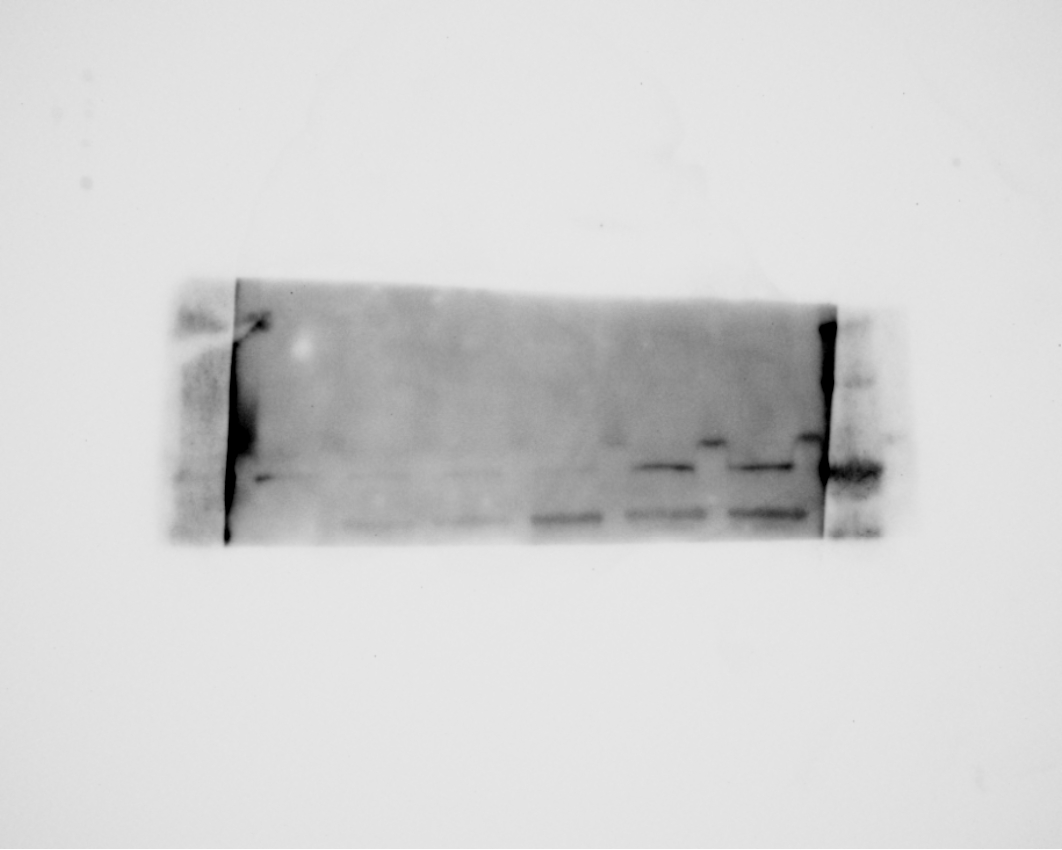

Supplement: Figure 5—figure supplement 1—source data 1. [file elife-71945-fig5-figsupp1-data1.zip › Figure 5 - figure supplement 1 - source data 1/Figure 5 - figure supplent 1 - source data 1_antiStrep.jpg]

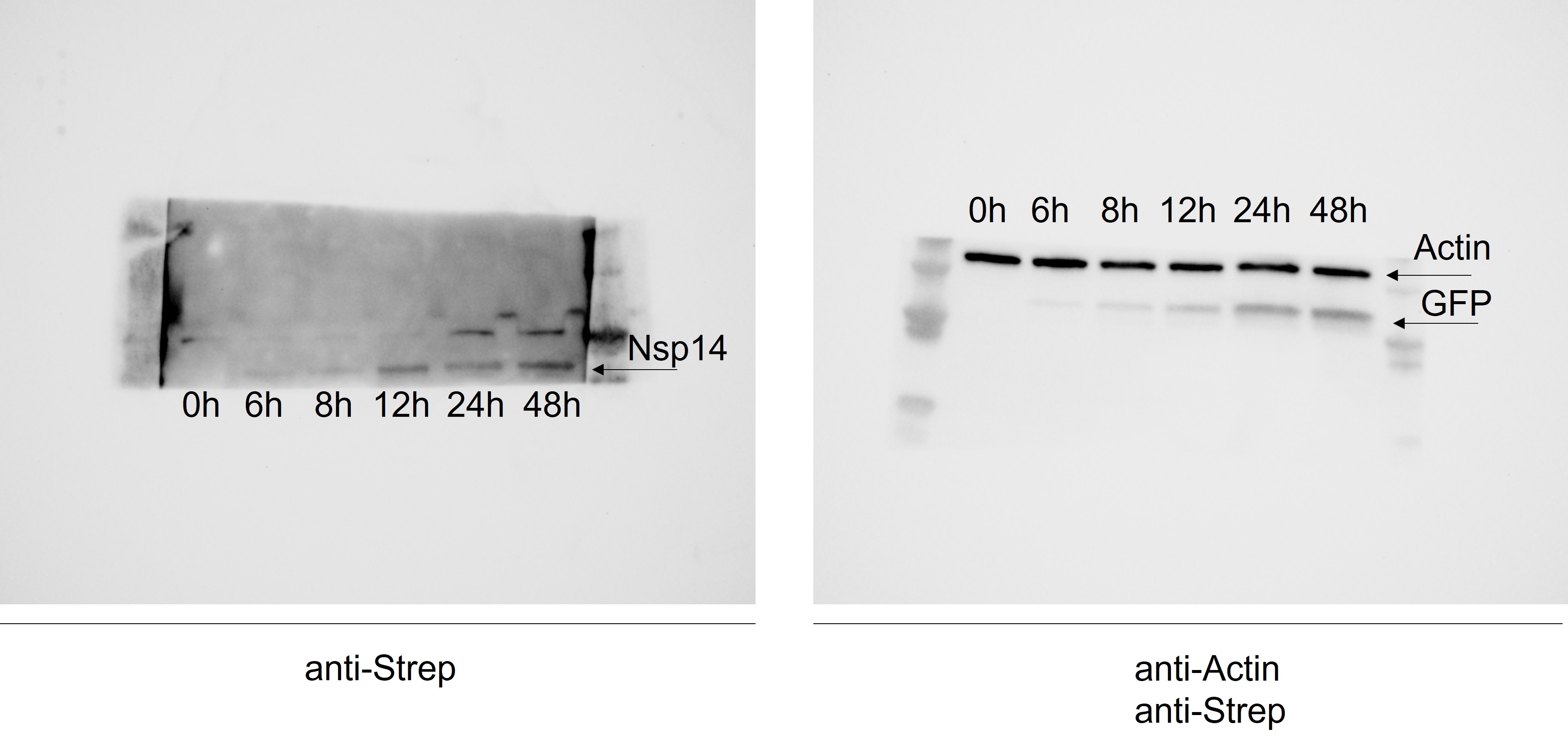

Supplement: Figure 5—figure supplement 1—source data 1. [file elife-71945-fig5-figsupp1-data1.zip › Figure 5 - figure supplement 1 - source data 1/Figure 5 - figure supplent 1 - source data 1_Labelled.jpg]

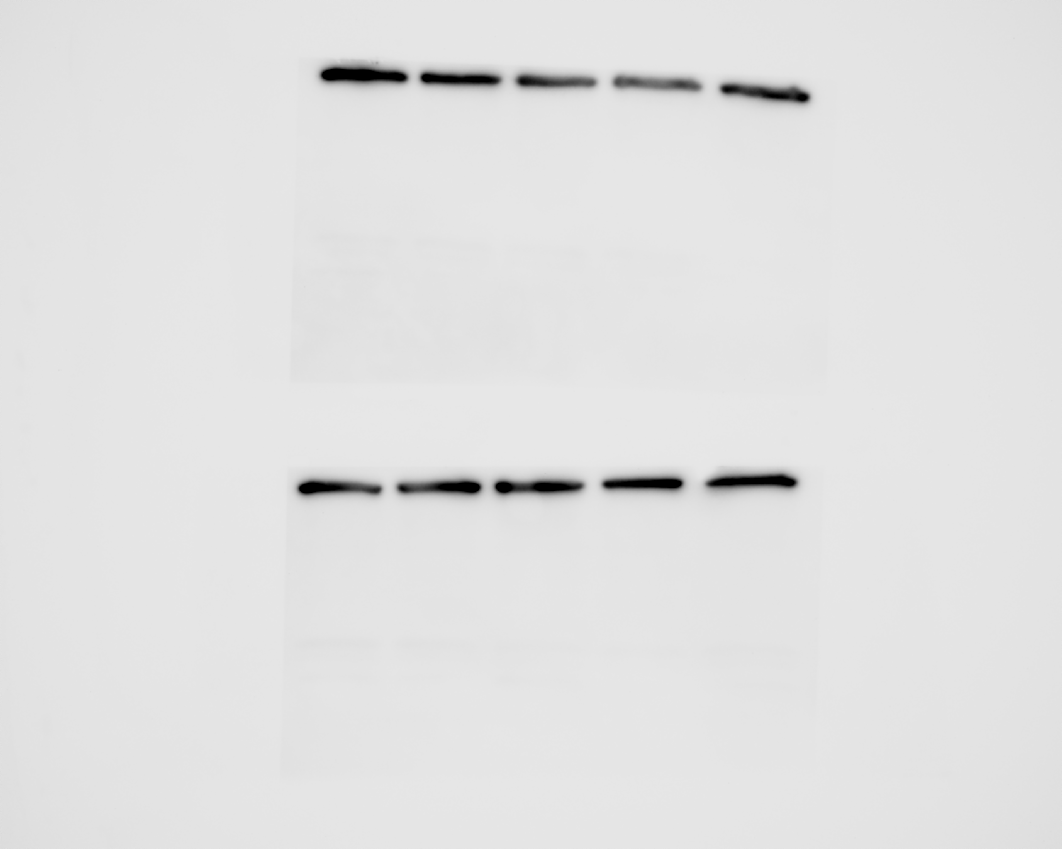

Supplement: Figure 6—source data 1. [file elife-71945-fig6-data1.zip › Figure 6 - source data 1/Figure 6 - source data 1_antiActin.tif]

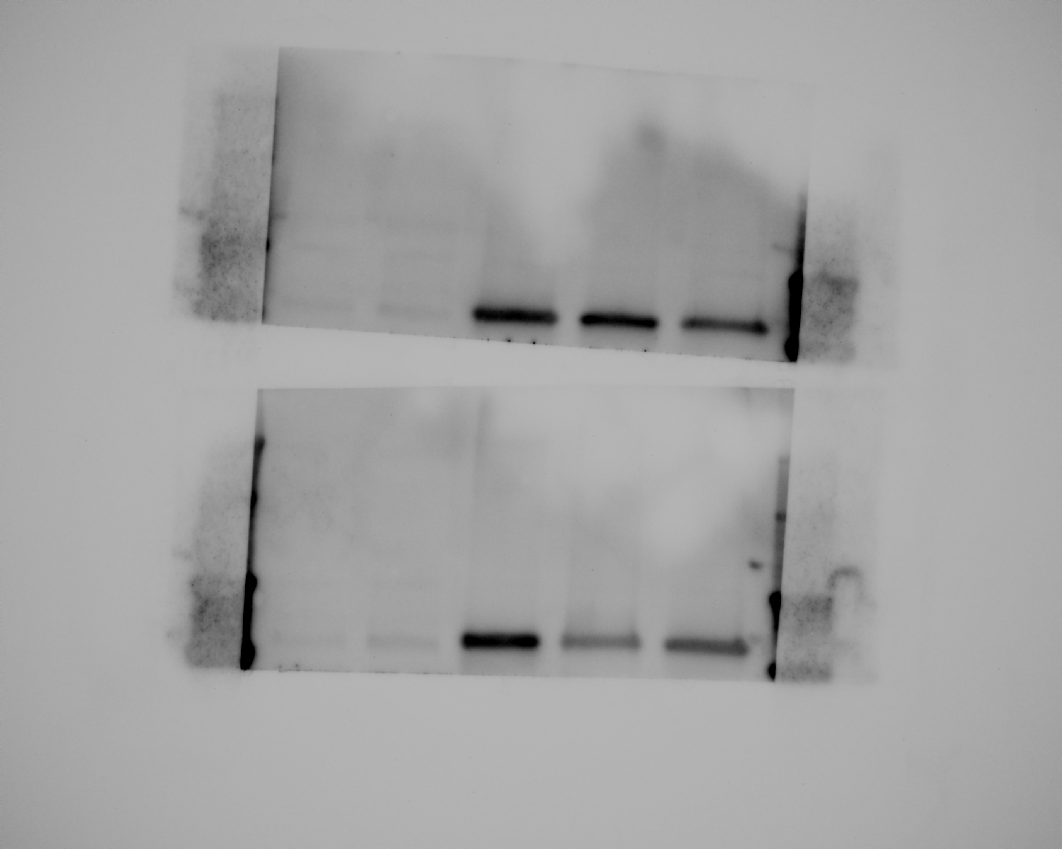

Supplement: Figure 6—source data 1. [file elife-71945-fig6-data1.zip › Figure 6 - source data 1/Figure 6 - source data 1_antiStrep.tif]

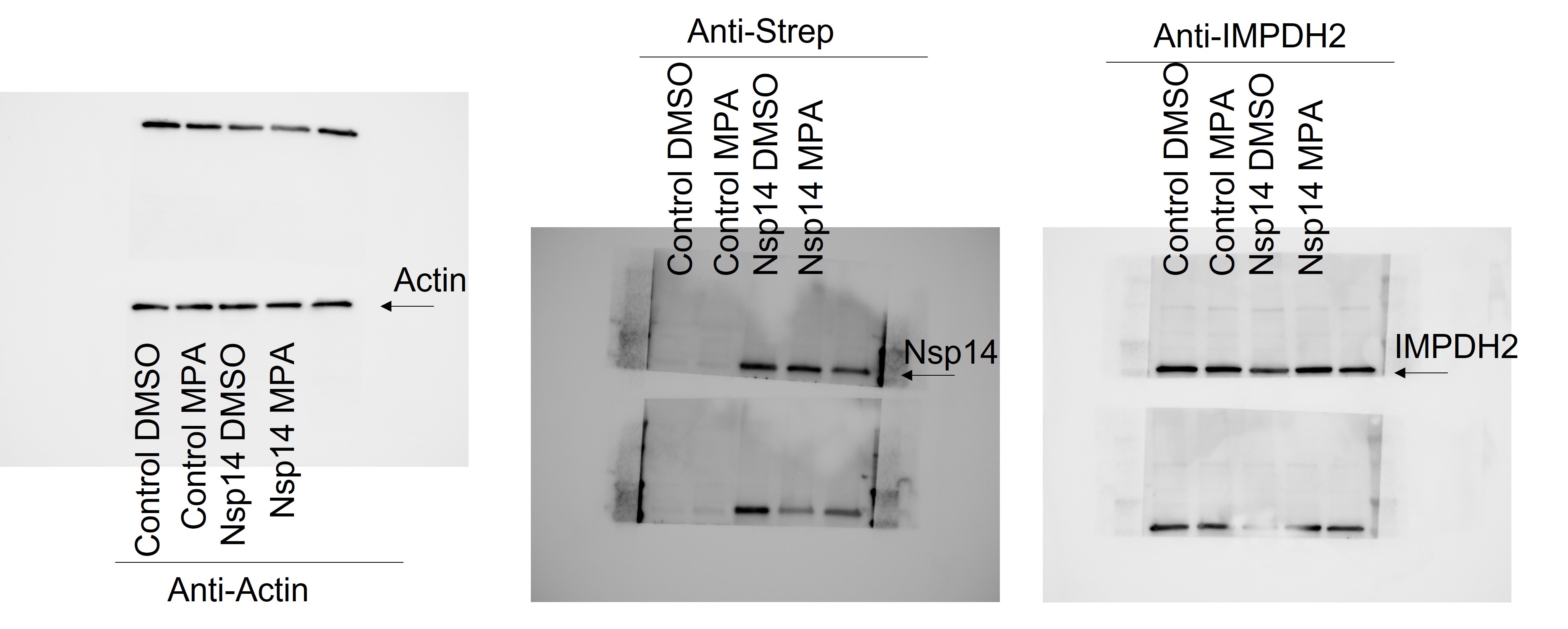

Supplement: Figure 6—source data 1. [file elife-71945-fig6-data1.zip › Figure 6 - source data 1/Figure 6 - source data 1_Labelled.jpg]

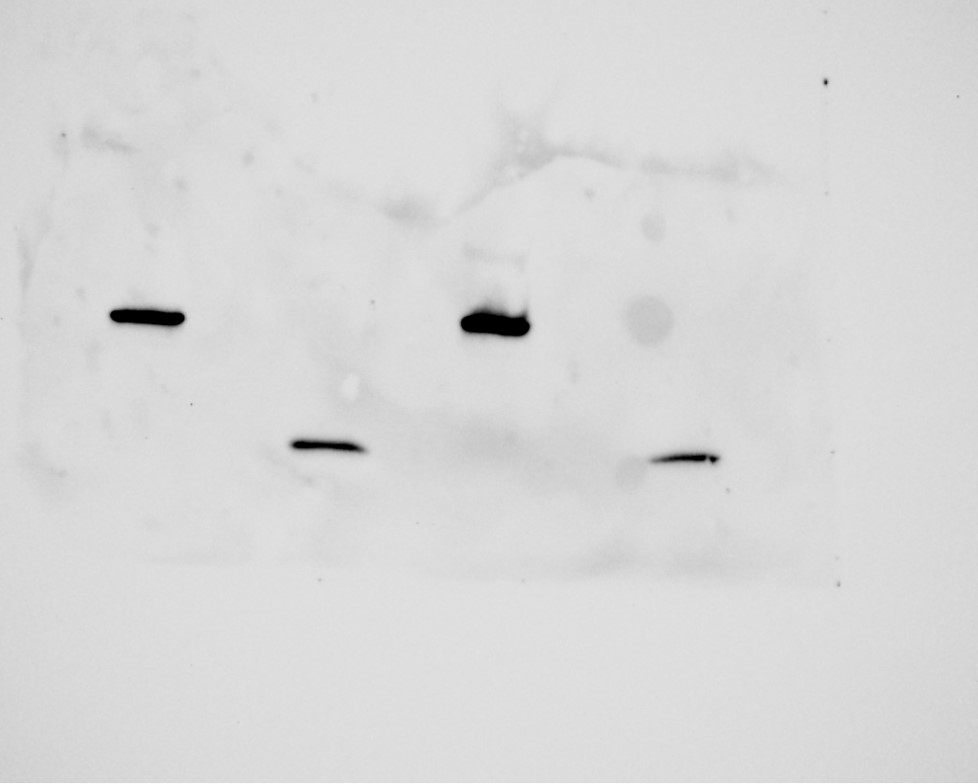

Supplement: Figure 6—figure supplement 1—source data 1. [file elife-71945-fig6-figsupp1-data1.zip › Figure 6 - figure supplement 1 - source data 1/Figure 6 - figure supplement 1 - source data 1_12h_antiGAPDH.jpg]

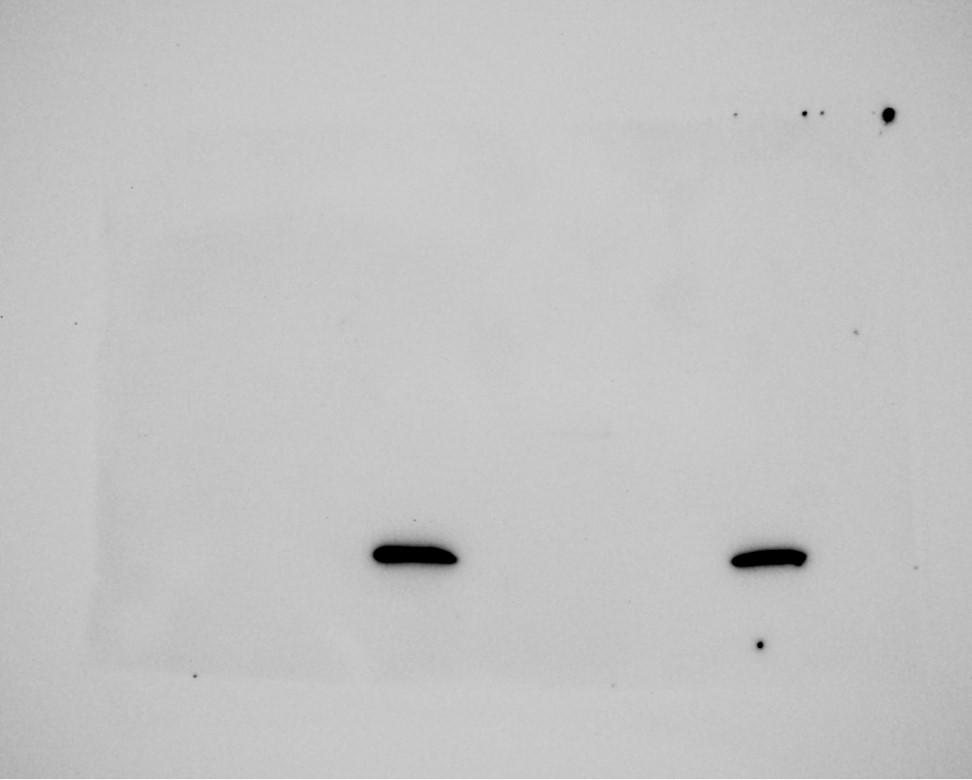

Supplement: Figure 6—figure supplement 1—source data 1. [file elife-71945-fig6-figsupp1-data1.zip › Figure 6 - figure supplement 1 - source data 1/Figure 6 - figure supplement 1 - source data 1_12h_antiH3K27me3.jpg]

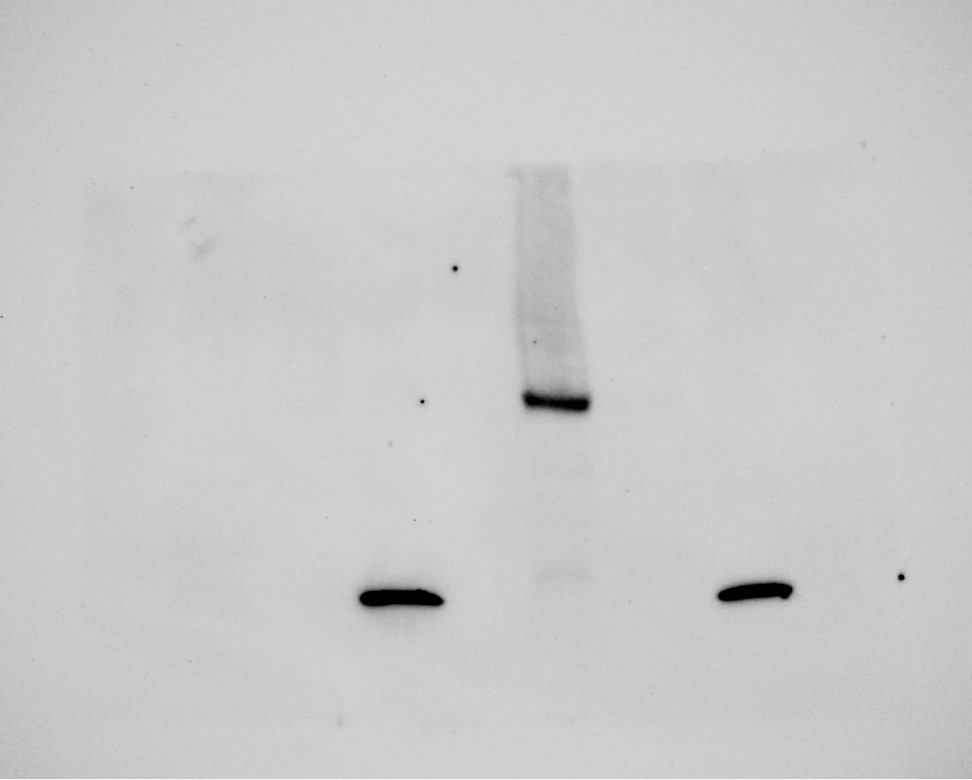

Supplement: Figure 6—figure supplement 1—source data 1. [file elife-71945-fig6-figsupp1-data1.zip › Figure 6 - figure supplement 1 - source data 1/Figure 6 - figure supplement 1 - source data 1_12h_antiStrep.jpg]

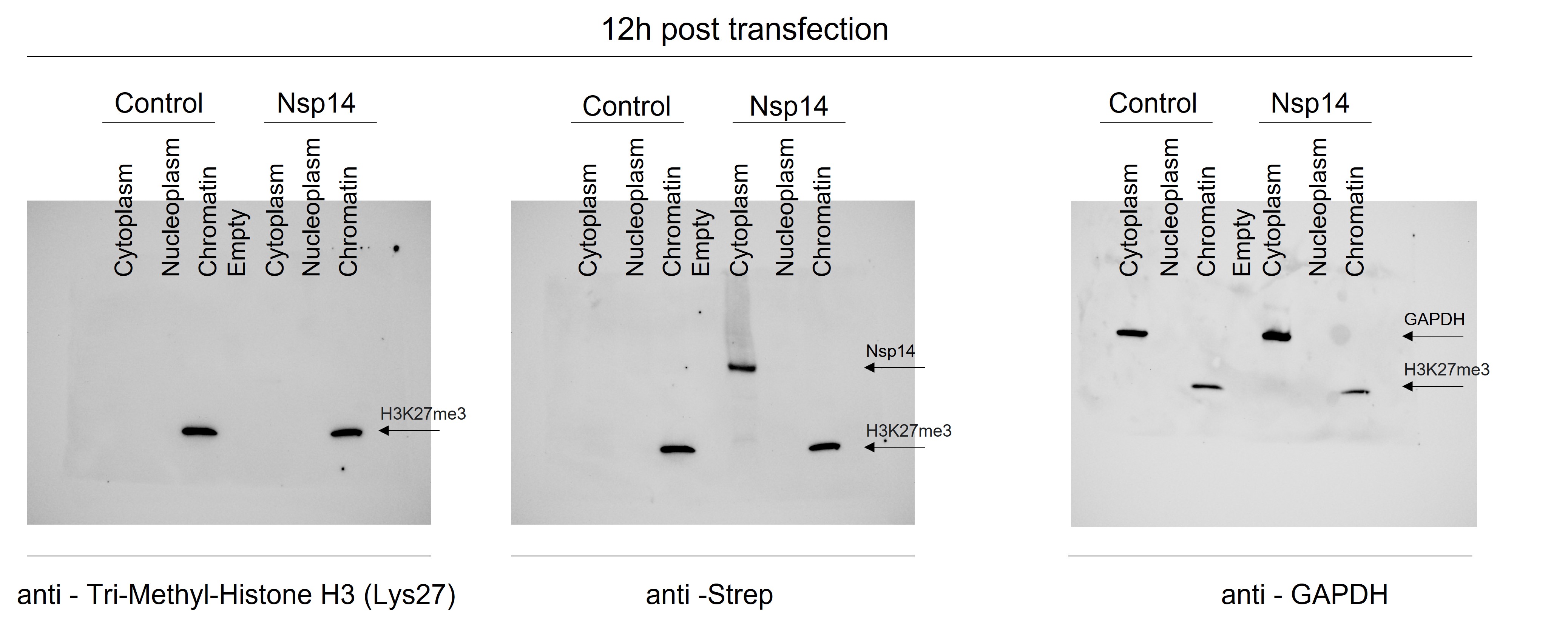

Supplement: Figure 6—figure supplement 1—source data 1. [file elife-71945-fig6-figsupp1-data1.zip › Figure 6 - figure supplement 1 - source data 1/Figure 6 - figure supplement 1 - source data 1_12h_Labelled.jpg]

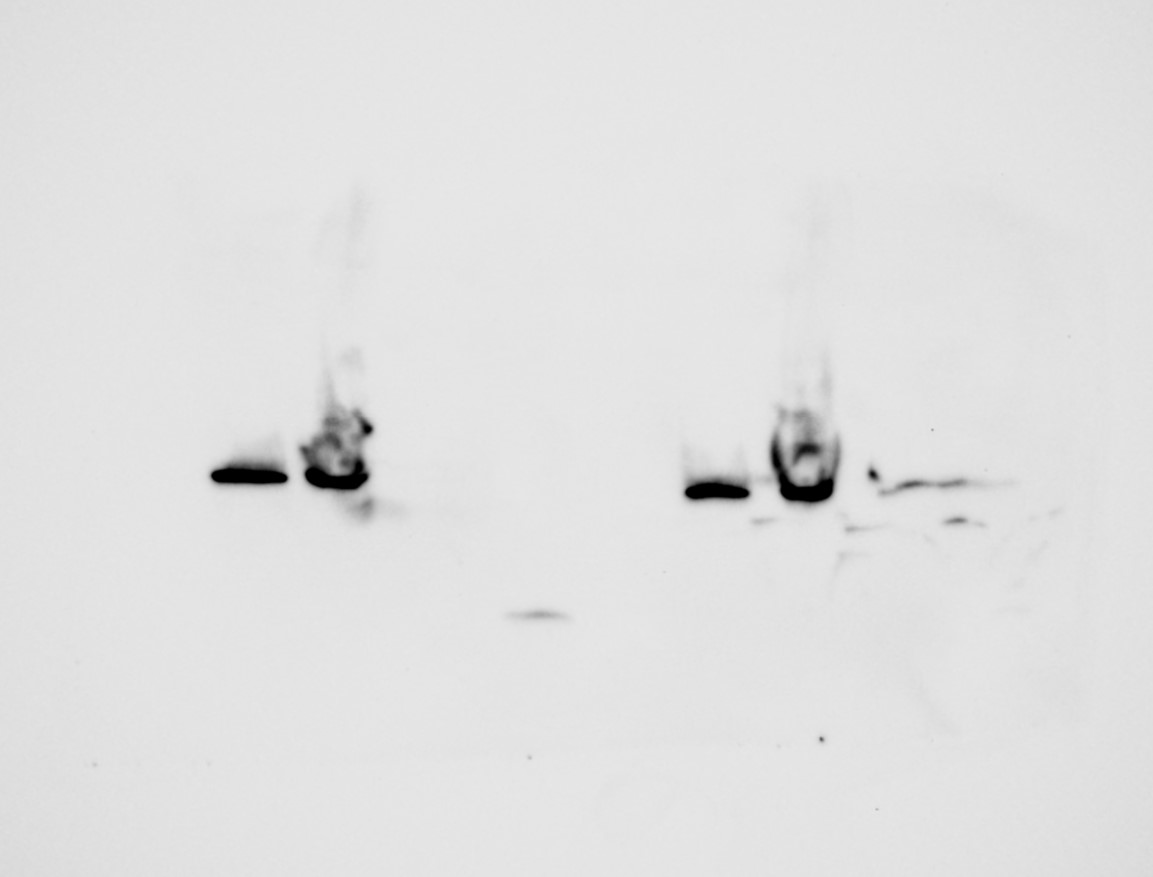

Supplement: Figure 6—figure supplement 1—source data 1. [file elife-71945-fig6-figsupp1-data1.zip › Figure 6 - figure supplement 1 - source data 1/Figure 6 - figure supplement 1 - source data 1_24h_antiGAPDH.jpg]

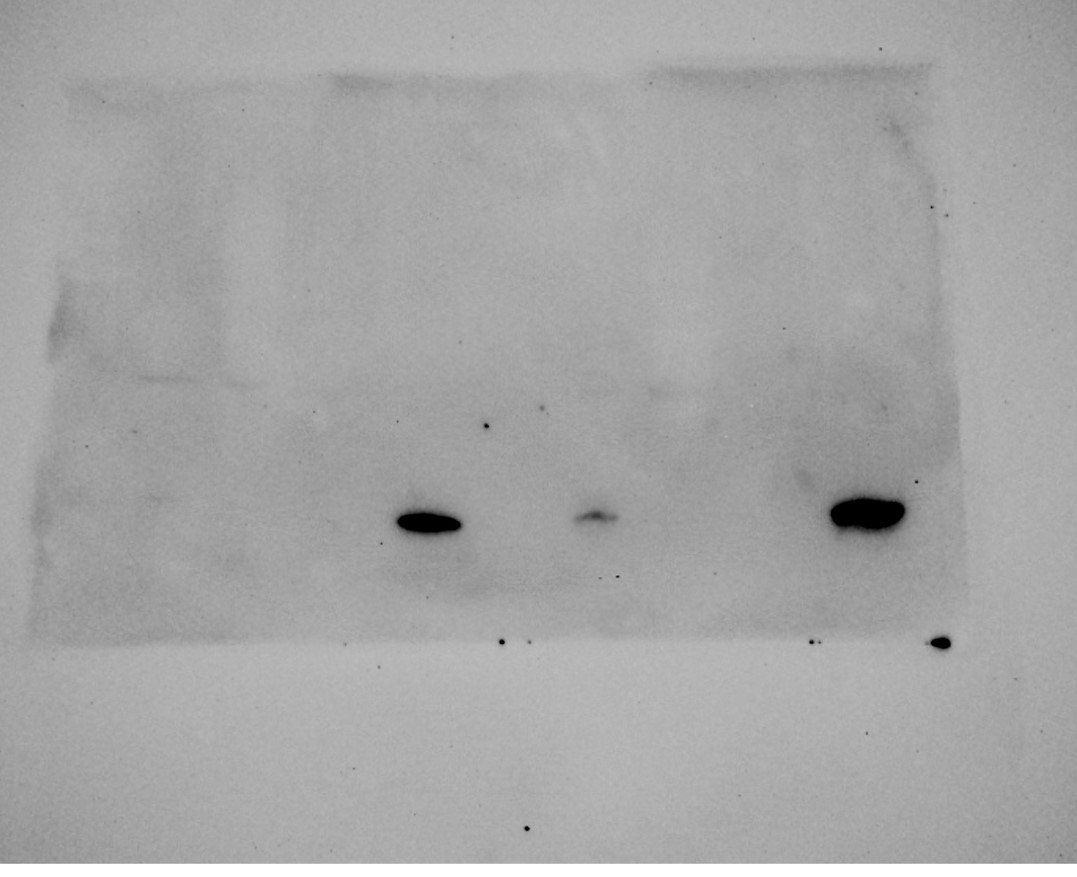

Supplement: Figure 6—figure supplement 1—source data 1. [file elife-71945-fig6-figsupp1-data1.zip › Figure 6 - figure supplement 1 - source data 1/Figure 6 - figure supplement 1 - source data 1_24h_antiH3K27me3.jpg]

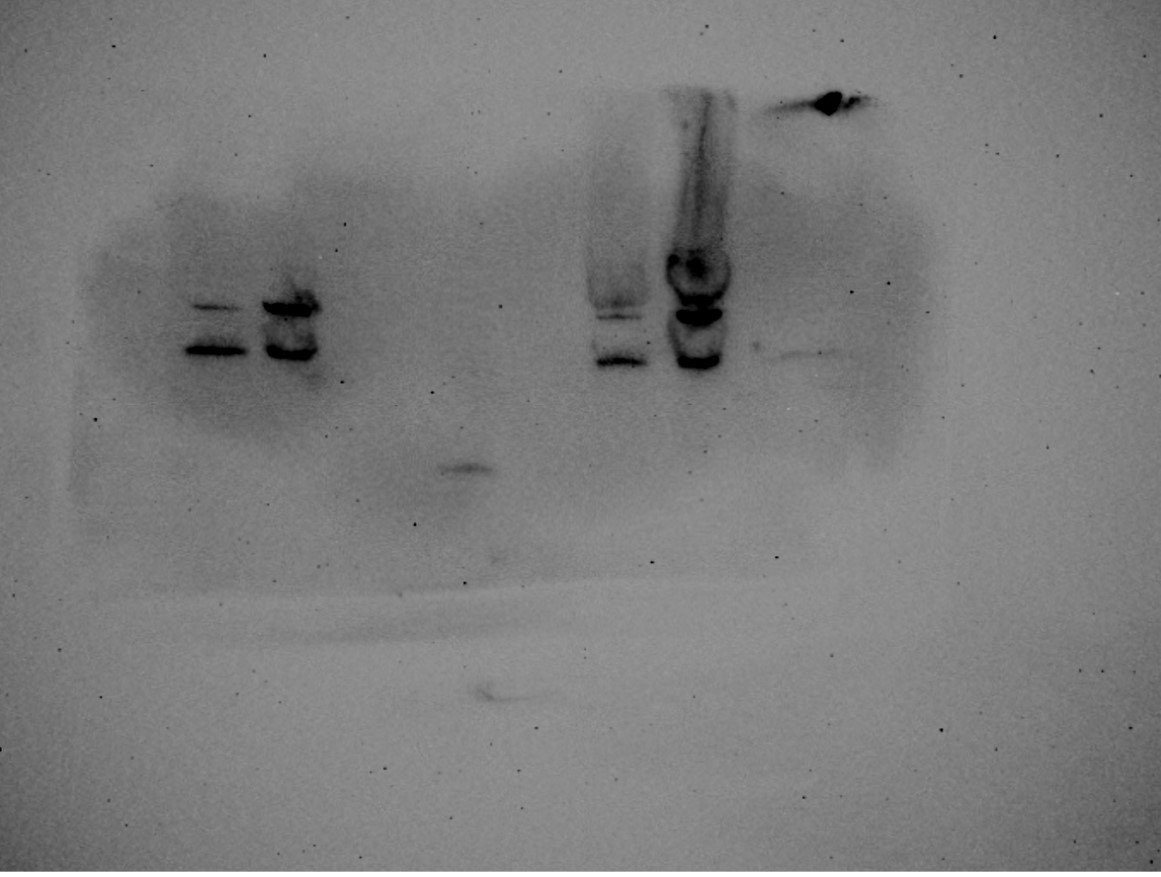

Supplement: Figure 6—figure supplement 1—source data 1. [file elife-71945-fig6-figsupp1-data1.zip › Figure 6 - figure supplement 1 - source data 1/Figure 6 - figure supplement 1 - source data 1_24h_antiStrep.jpg]

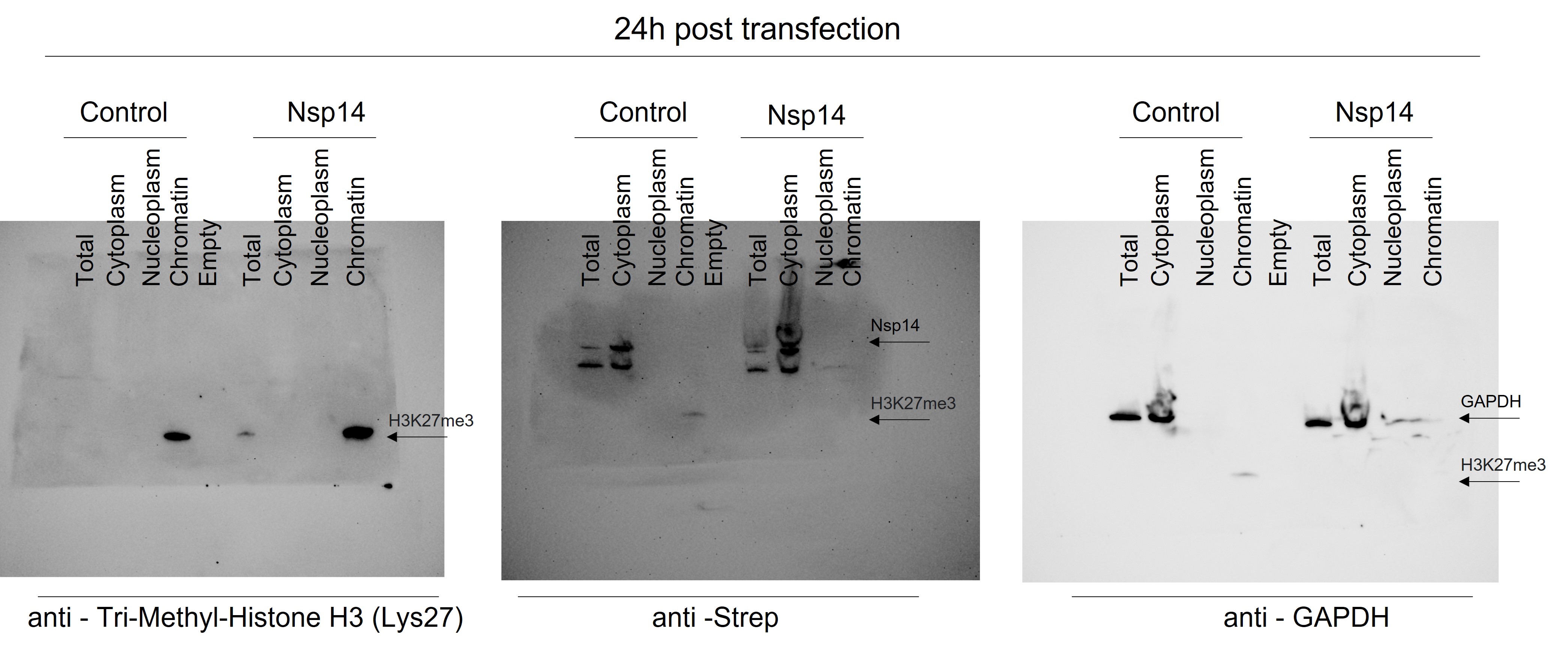

Supplement: Figure 6—figure supplement 1—source data 1. [file elife-71945-fig6-figsupp1-data1.zip › Figure 6 - figure supplement 1 - source data 1/Figure 6 - figure supplement 1 - source data 1_24h_Labelled.jpg]

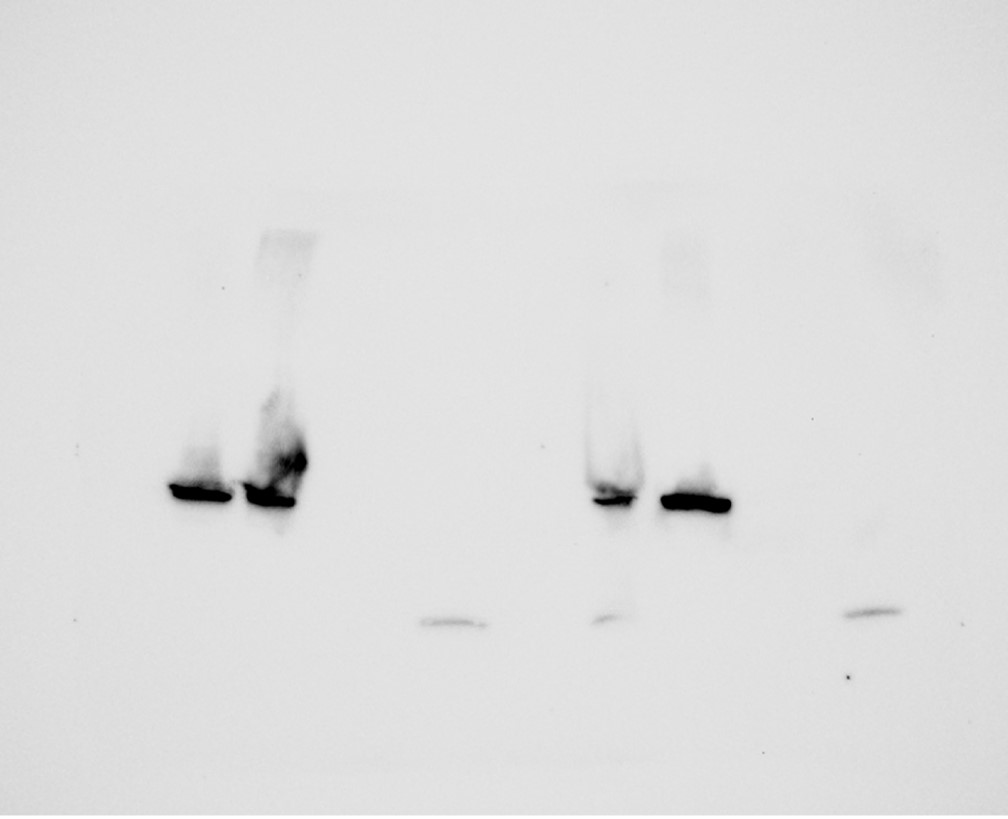

Supplement: Figure 6—figure supplement 1—source data 1. [file elife-71945-fig6-figsupp1-data1.zip › Figure 6 - figure supplement 1 - source data 1/Figure 6 - figure supplement 1 - source data 1_48h_antiGAPDH.jpg]

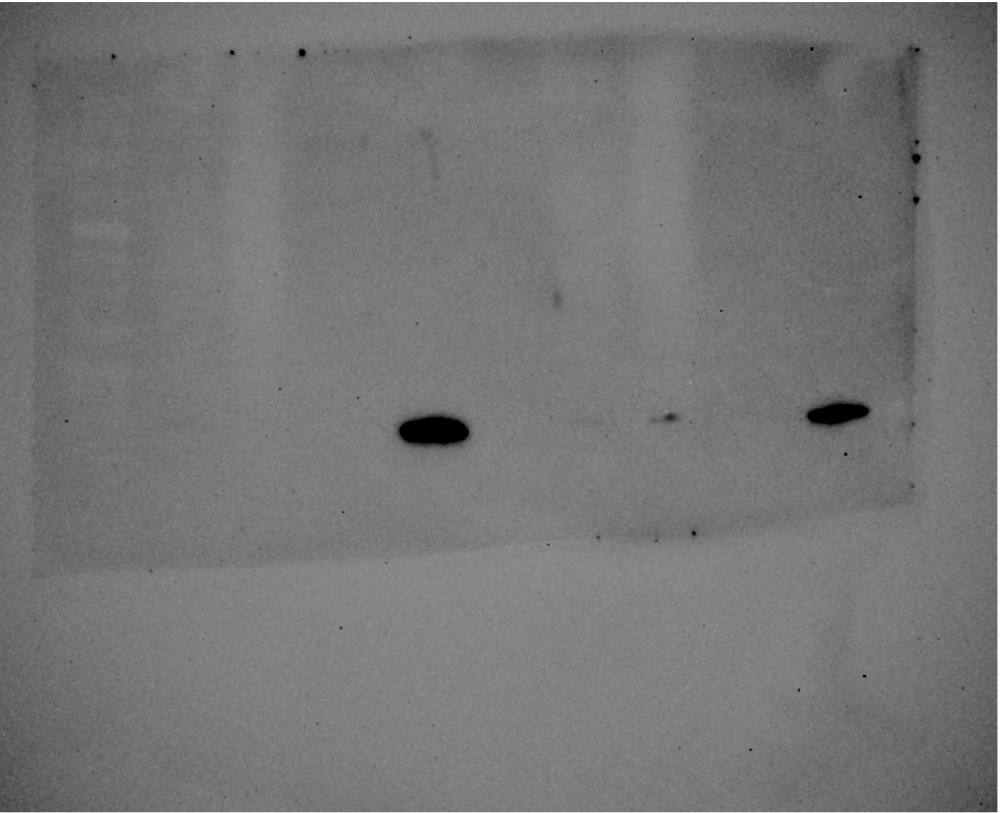

Supplement: Figure 6—figure supplement 1—source data 1. [file elife-71945-fig6-figsupp1-data1.zip › Figure 6 - figure supplement 1 - source data 1/Figure 6 - figure supplement 1 - source data 1_48h_antiH3K27me3.jpg]

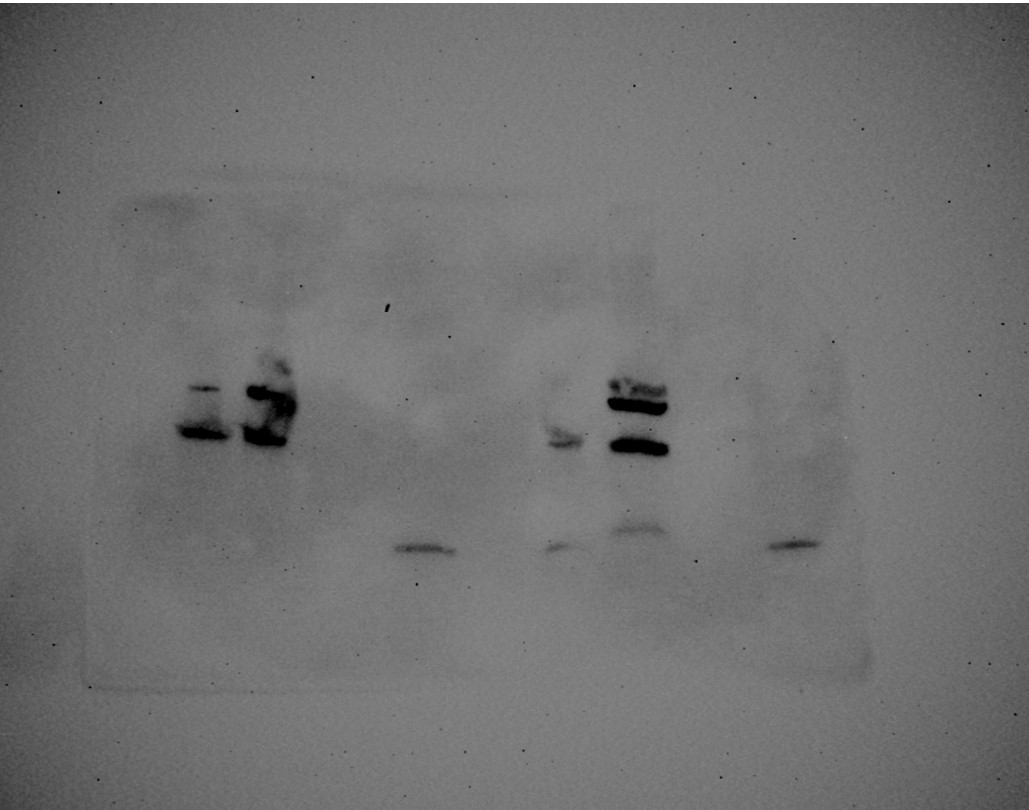

Supplement: Figure 6—figure supplement 1—source data 1. [file elife-71945-fig6-figsupp1-data1.zip › Figure 6 - figure supplement 1 - source data 1/Figure 6 - figure supplement 1 - source data 1_48h_antiStrep.jpg]

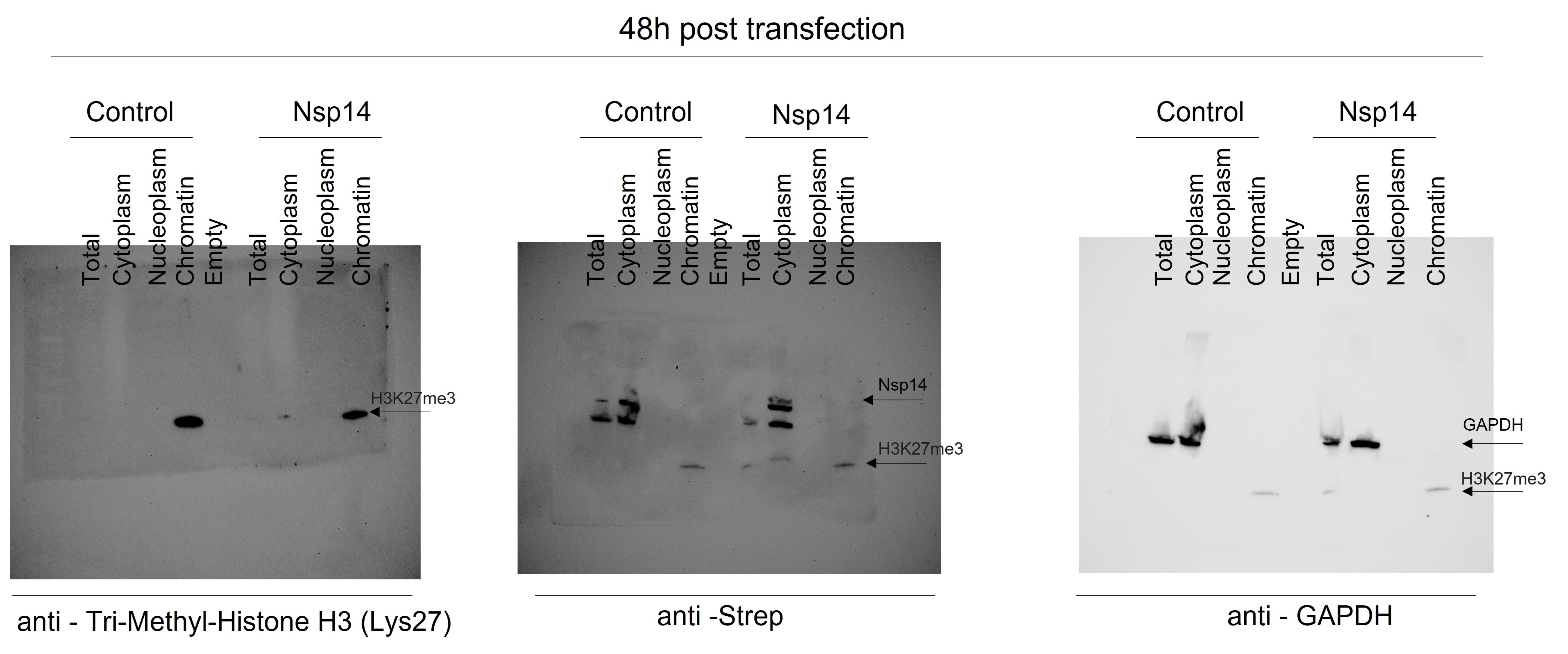

Supplement: Figure 6—figure supplement 1—source data 1. [file elife-71945-fig6-figsupp1-data1.zip › Figure 6 - figure supplement 1 - source data 1/Figure 6 - figure supplement 1 - source data 1_48h_Labelled.jpg]

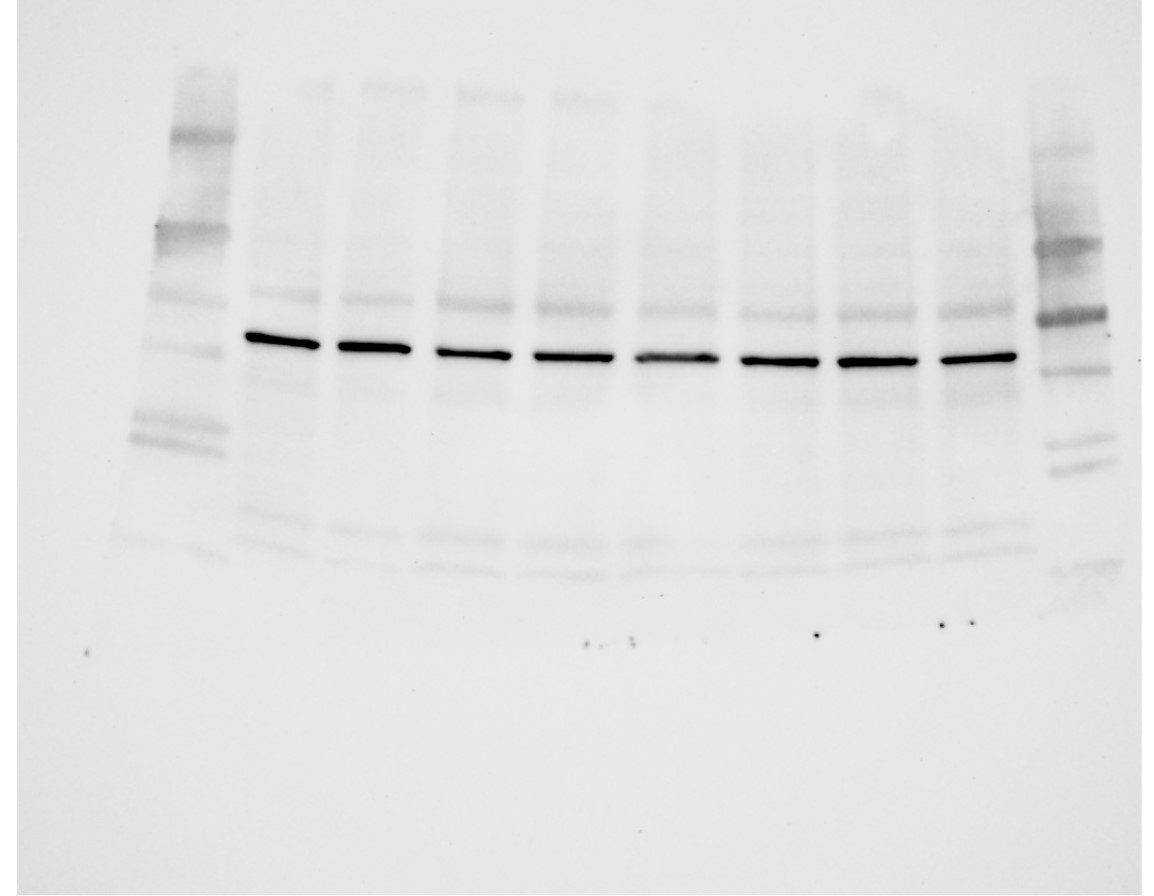

Supplement: Figure 6—figure supplement 2—source data 1. [file elife-71945-fig6-figsupp2-data1.zip › Figure 6 - figure supplement 2 - source data 1/Figure 6 - figure supplement 2 - source data 1_AntiActin.jpg]

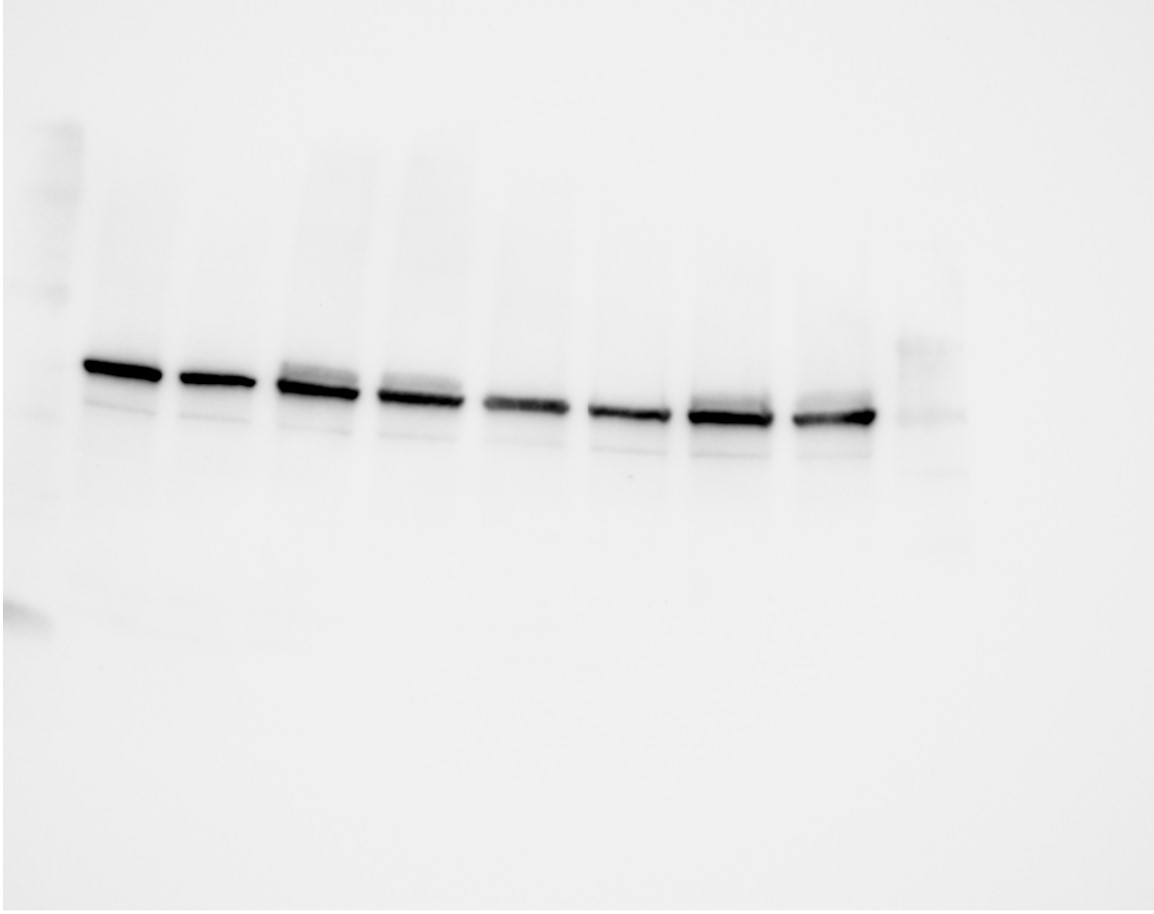

Supplement: Figure 6—figure supplement 2—source data 1. [file elife-71945-fig6-figsupp2-data1.zip › Figure 6 - figure supplement 2 - source data 1/Figure 6 - figure supplement 2 - source data 1_AntiIMPDH2.jpg]

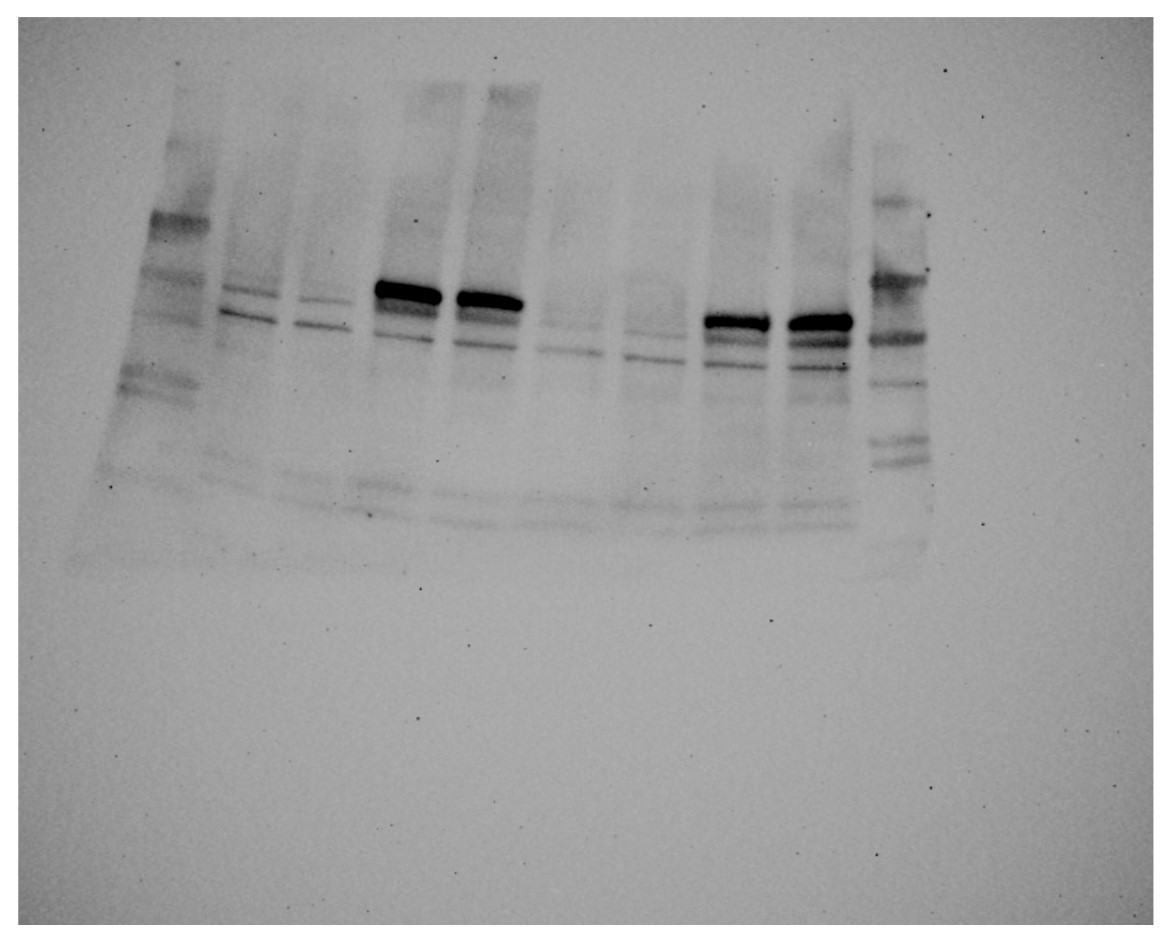

Supplement: Figure 6—figure supplement 2—source data 1. [file elife-71945-fig6-figsupp2-data1.zip › Figure 6 - figure supplement 2 - source data 1/Figure 6 - figure supplement 2 - source data 1_AntiNsp14.jpg]

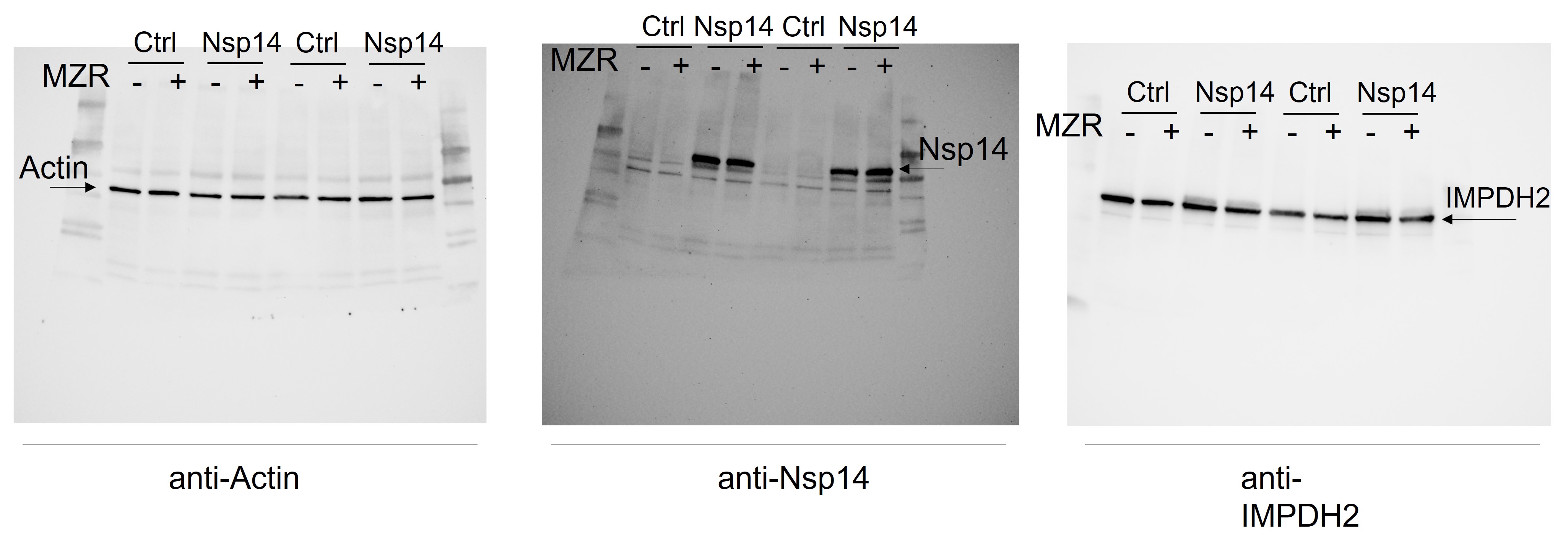

Supplement: Figure 6—figure supplement 2—source data 1. [file elife-71945-fig6-figsupp2-data1.zip › Figure 6 - figure supplement 2 - source data 1/Figure 6 - figure supplement 2 - source data 1_Labelled.jpg]
